# Inhibiting vascular smooth muscle cell proliferation mediated by osteopontin *via* regulating gut microbial lipopolysaccharide: A novel mechanism for paeonol in atherosclerosis treatment

**DOI:** 10.3389/fphar.2022.936677

**Published:** 2022-08-11

**Authors:** Xiaoyan Shi, Hongfei Wu, Yarong Liu, Hanwen Huang, Ling Liu, Yulong Yang, Tingting Jiang, Min Zhou, Min Dai

**Affiliations:** ^1^ College of Pharmacy, Anhui University of Chinese Medicine, Hefei, China; ^2^ Anhui Key Laboratory for Research and Development of Traditional Chinese Medicine, Hefei, China

**Keywords:** gut microbiota, lipopolysaccharide, osteopontin, atherosclerosis, paeonol

## Abstract

**Background:** Although the gut microbiota is involved in metabolic disease such as atherosclerosis, the underlying mechanism remains elusive. Paeonol (Pae) is a natural phenolic compound isolated from Cortex Moutan, which exhibits anti-atherosclerotic effects. Our previous research demonstrated gut microbiota as a site of Pae action. However, the mechanism by which Pae exerts its anti-atherosclerotic effect by the regulation of gut microbiota remains unclear.

**Objective:** To investigate a potential mechanistic link between the gut microbial lipopolysaccharide (LPS) and vascular smooth muscle cell (VSMC) proliferation in atherosclerosis progression and explore the possible role of Pae.

**Methods:** Experimental atherosclerosis was established in ApoE^−/−^ mice, and the atherosclerosis mice were treated with Pae for 4 weeks before being sacrificed for analyses while conducting fecal microbiota transplantation (FMT). The plaque area, levels of serum LPS, expressions of inflammatory factors in serum or aorta, and intestinal barrier permeability were determined. VSMCs were co-cultured with THP-1 cells. CCK-8 assay and EdU staining were performed to assess the proliferative capacity of VSMCs. Immunofluorescence staining was performed to observe the nuclear transfer of p65. Western blotting was used to detect the candidate protein expression level, and quantitative real-time PCR (qRT-PCR) was used to detect the mRNA expression level in tissues or cells of each group.

**Results:** During atherosclerosis progression, gut dysbiosis leads to the peripheral accumulation of gut microbial LPS, which acts as a trigger to stimulate osteopontin (OPN) production from circulating monocytes, inducing cell-to-cell crosstalk to promote VSMC proliferation in the aorta. Importantly, the elevation of LPS and OPN concentrations in the blood was also observed in patients with atherosclerosis. Pae could significantly improve atherosclerosis, suppress gut microbial LPS accumulation, and inhibit monocyte/macrophage activation and VSMC proliferation.

**Conclusions:** The present study provides a mechanistic scenario for how long-term stimulation of gut microbial LPS in circulating blood generates a pathological secondary response that leads to abnormal proliferation of VSMCs using high OPN expression in circulating monocytes and suggests a novel strategy for atherosclerosis therapy by remodeling the gut microbiota.

## Graphical Abstract

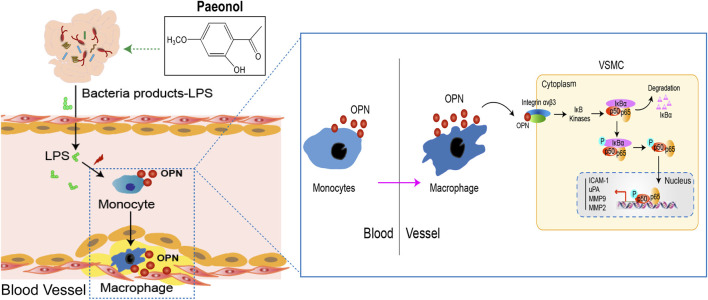

## Introduction

Atherosclerosis is a chronic inflammatory disease mediated by the interaction of multiple cells (Hansson et al., 2006; Wolf and Ley, 2019). Despite advancements in research efforts, the molecular mechanisms underlying the onset and progression of atherosclerosis remain unclear. Recently, it has been highlighted that gut microbiota has direct effects on the inflammatory reaction (Wastyk et al., 2021), playing a critical role in regulating chronic inflammatory disease, including atherosclerosis. Dysbiosis of the gut microbiota may affect the progression of atherosclerosis remotely by producing metabolites that increase the inflammatory response (Wang et al., 2015; Tang et al., 2017). In particular, a high circulating level of gut microbial lipopolysaccharide (LPS) was found in atherosclerosis patients (Loffredo et al., 2020), and LPS could promote the inflammatory proliferation of vascular smooth muscle cells (VSMCs). At present, the understanding of the mechanistic link between gut microbiota and VSMCs proliferation in atherosclerosis progression is still very limited.

Communication between peripheral blood cells and resident cells in the aortic wall is generally mediated by a number of cytokines that regulate atherosclerosis (Economou et al., 2015; Yu et al., 2019). Crosstalk between monocytes/macrophages and VSMC plays an important role in the process of atherosclerosis (Zhu et al., 2019). Importantly, these two cell types regulate the proliferation of VSMCs by communicating with each other through the classical secretion of soluble factors, such as inflammatory cytokines. Osteopontin (OPN, encoded by Spp1), a secreted proinflammatory protein involved in the proliferation of VSMCs, is an important driver of atherosclerosis (Sun et al., 2009; Zhang et al., 2013). Recent studies have shown that acute *in vivo* LPS stimulation enhances OPN expression in mouse peripheral blood monocytes (Fatkhullina et al., 2018). The underlying driving force and molecular mechanism that pre-activated the circulating monocytes recruited to the vascular wall, leading to the proliferation of VSMCs, remains obscure.

Paeonol (Pae), a bioactive compound extracted from the root of *Paeonia albiflora*, which has a long history of clinical application as a potential anti-inflammatory agent. At present, the dosage forms of Pae approved by China Food and Drug Administration include tablet, ointment, and adhesive plaster (Zhang et al., 2019). Our previous studies have shown that Pae exerts antiatherosclerotic effect by alleviating inflammation and inhibits abnormal proliferation of VSMCs in the aorta (Wu et al., 2017; Liu et al., 2018). Furthermore, Pae treatment remarkably inhibited atherosclerosis progression in HFD-fed ApoE^−/−^ mice *via* altering the composition of gut microbiota (unpublished data). However, the underlying mechanisms remain unclear.

In the present study, we aimed to investigate the mechanistic relationship between gut microbial LPS and VSMC proliferation and to explore potential treatment strategies. Here, we have revealed that gut microbial LPS upregulated pro-atherogenic OPN expression in circulating monocytes, which drives the proliferation of VSMCs during atherosclerosis progression. Using pharmacological approaches, we proposed a novel mechanism for the regulation of VSMC proliferation by the gut microbial LPS-triggered gut-vascular axis, which remotely controls the progression of atherosclerosis. Pae particularly inhibited the microbial LPS biosynthesis pathway, decreased the abnormal production of LPS and OPN-mediated crosstalk between THP-1 monocytes and VSMCs, and in turn controlled VSMC proliferation. We suggest that the decrease of gut microbial LPS induced by Pae is an important contributor in inhibiting VSMC proliferation.

## Materials and methods

### Ethics statement

All surgical and experimental procedures were approved by the Ethics Committee of the Anhui University of Chinese Medicine. The protocol of human tests was approved by the medical research ethics committee of the First Affiliated Hospital of Anhui University of Chinese Medicine.

### Patients

Patients with newly diagnosed atherosclerosis (*n* = 15) were recruited from inpatients of the First Clinical Medical School of Anhui University of Chinese Medicine between October 2020 and November 2020. All patients gave informed written consent to participate. All the enrolled patients were diagnosed with obvious carotid atherosclerosis with sclerotic plaque formation by carotid ultrasound. In addition, healthy volunteers (*n* = 15) were selected as the control group. The study was approved by the First Clinical Medical School of Anhui University of Chinese Medicine.

### Animal and atherosclerosis models

All animal experiments were approved by the Institutional Animal Care and Use Committee of Anhui University of Chinese Medicine. ApoE^−/−^ mice were purchased from Cavens Laboratory Animal Co., Ltd. (Changzhou, China), and the atherosclerosis mouse model was replicated as described in our previous study. All the male ApoE^−/−^mice were housed in temperature-controlled cages (23°C–25°C) under a 12/12-h light/dark cycle with free access to water and fed a high-fat diet (HFD, containing 21% fat and 0.15% cholesterol) until atherosclerosis lesions were obviously formed on arteries.

After 12°weeks of HFD feed modeling, all the ApoE^−/−^ mice were randomly divided into three groups: the model group, medium-dose Pae group (200 mg/kg), and high-dose Pae group (400 mg/kg). Briefly, atherosclerosis animals were orally administered with Pae (99% purity) in contained 5% CMC-Na solution daily for total 4°weeks. At the same time, C57BL/6J mice received water during the experiment as the control group. Biological samples, including serum, aorta, and colon, of mice were collected after euthanasia.

### Plaque lesion analysis

As mentioned earlier, we used Oil Red O (ORO) staining and hematoxylin and eosin (H&E) staining assay to detect the atherosclerotic lesions in the aorta. The entire artery, including the aortic arch, thoracic, and abdominal regions, was carefully dissected longitudinally along the midline, followed by ORO staining. For H&E staining, we processed the aortic tissue into paraffin-embedded blocks to produce 5-µm thick sections and placed on glass slides for subsequent staining steps. The percentage of the lesion area was determined by Image-Pro Plus 6.0 software.

### Antibiotic treatment and microbiota transfer experiments

Atherosclerosis mice were treated with water containing a mix of broad-spectrum antibiotics: ampicillin 1 g/L, metronidazole 0.5 g/L, vancomycin 0.5 g/L, and neomycin 1 g/L. Microbiota recipients were maintained for 8°days with a mix of broad-spectrum antibiotics in drinking water. Microbiota from the cecum of atherosclerosis mice fed with HFD or Pae was collected, weighted, and resuspended at a concentration of 100 mg/ml in PBS. Transplantation was performed by an oral gavage of 200 μl transplant material once per week.

### Lipopolysaccharide supplementation

In the beginning, at 12 weeks of age, ApoE^−/−^ mice were administered i.p. with LPS (50 mg per injection) every 3 days for additional 4 weeks of ND feeding. After that, changes in atherosclerosis progression and inflammatory gene expression were analyzed.

### Detection of serum lipopolysaccharide levels

The level of serum LPS was measured using an enzyme-linked immunosorbent assay (ELISA) kit based on the manufacturer’s instructions. Briefly, the serum sample to be tested and the standard were added to the corresponding wells and incubated for 90 min at 37°C in the dark. The liquid in the plate was then discarded, and 300 μl of wash buffer was added to each well and discarded after standing for 30 s. Next, 100 μl of biotin antibody working solution was slowly added to each well and incubated at 37°C for 60 min in the dark. After that reaction was finished, it was repeated five times, slowly adding 100 μl of the enzyme-combined working solution, incubated at 37°C for 30 min, washing it five times again, adding a color developing agent, and the optical density was measured at 450 nm using a microplate reader.

### Alcian blue staining

Wax blocks of colon tissue from each group of mice were sectioned at 5 μm thickness, followed by staining using an Alcian blue staining kit, according to the manufacturer’s instructions.

### Cell culture

VSMCs were isolated and primarily cultured from C57BL/6J mouse arteries, as described in our previous study (Wu et al., 2017). It should be noted that the VSMCs used in the experiment are all within passage 10. Human THP-1 cells were purchased from Procell Life Science&Technology Co., Ltd (Wuhan, China). THP-1 cells were cultured in RPMI 1640 medium containing 10% FBS (Gibco BRL, Grand Island, United States) in a humidified atmosphere of 5% CO_2_ at 37°C.

### Co-culture system of THP-1 cells and vascular smooth muscle cells

A co-culture system of THP-1 cells and VSMCs was established using Transwell membranes (pores 0.4 μm, Merck Millipore, United States). VSMCs were plated on the bottom of a 6-well cell culture plate, THP-1 cells were seeded on the upper transwell membrane, experiments were performed after overnight culture, and VSMCs were collected separately for further manipulation.

### Cell proliferation assay

The ability of VSMCs proliferation was evaluated using CCK-8 assay and EdU incorporation assay. First, VSMCs were seeded in 96-well plates and treated with various concentrations of rhOPN for 24 h, followed by the addition of 10 μl of CCK-8 solution. After 1 h incubation, the absorbance was measured spectrophotometrically at 450 nm. Second, VSMCs were cultured in 24-well plates and treated with 0.5 μm rhOPN for 24 h. The proliferation of VSMCs was then observed by EdU staining, according to the manufacturer’s instructions. The nuclei were stained with DAPI for 10 min and finally observed by fluorescence microscopy.

### Immunostaining of p65

VSMCs previously seeded in 24-well plates and subjected to intervention stimulation were incubated overnight with primary antibody at 4°C, followed by addition of fluorescently labeled secondary antibody. Nuclear transfer of p65 was observed by fluorescence microscopy (Leica, Germany).

### Quantitative real-time PCR (qRT-PCR)

Initially, TRIzol reagent (Invitrogen) was used to isolate total RNA from mouse aortic tissues or cultured cells. After chloroform was added to the mixture, the mixture was vortexed sufficiently, and RNA in the upper aqueous phase was taken after centrifugation. An equal amount of isopropanol was then added to precipitate the RNA. The obtained RNA was washed with 75% ethanol and dissolved in DEPC water. After the purity of the extracted RNA was determined to be satisfactory, it was reverse-transcribed using a PrimeScript 1st Strand cDNA Synthesis kit (TaKaRa). qRT-PCR was then performed in the StepOnePlus Real-Time PCR System (Agilent MX3000P real-time PCR detection system). GAPDH was served as endogenous control. The mouse-specific primers used in the qRT-PCR reaction are listed in Table1.

**TABLE 1:** PCR gene-specific primer pairs.

Gene	Primer pair (5′-3′)
OPN	Fw: TGG​CTA​TAG​GAT​CTG​GGT​GC
	Rw: ATT​TGC​TTT​TGC​CTG​TTT​GG
ICAM-1	Fw: CAA​TTT​CTC​ATG​CCG​CAC​AG
	Rw: AGC​TGG​AAG​ATC​GAA​AGT​CCG
MMP2	Fw: GAC​CAT​GCG​GAA​GCC​AAG​A
	Rw: TGT​GTA​ACC​AAT​GAT​CCT​GTA​TGT​G
MMP9	Fw: TTC​GCA​GAC​CAA​GAG​GGT​TTT​C
	Rw: AAG​ATG​TCG​TGT​GAG​TTC​CAG​GGC
β-actin	Fw: CTG​TAT​GCC​TCT​GGT​CGT​AC
	Rw: TGA​TGT​CAC​GCA​CGA​TTT​CC

Fw, forward primer; Rw, reverse primer.

### Western blot analysis

Total proteins were extracted from animal tissues and cells with RIPA buffer (Beyotime), containing 1% protease and phosphatase inhibitors, respectively. The extracted total protein was quantified and then subjected to electrophoresis, followed by Western blotting with rabbit polyclonal antibody against α-SMA (ab5694, 1:1,000, Abcam, Cambridge, MA, United States), rabbit monoclonal antibody against PCNA (ab92552, 1:2000, Abcam), rabbit monoclonal antibody against claudin-1 (ab180158, 1:2000, Abcam), rabbit monoclonal antibody against occludin (ab167161, 1:1,000, Abcam), rabbit monoclonal antibody against ZO-1 (ab576131, 1:1,000, Abcam), rabbit polyclonal antibody against OPN (ab283656, 1:1,000, Abcam), rabbit monoclonal antibody against p65 (ab32536, 1:1,000, Abcam), rabbit polyclonal antibody against p-p65 (Ser486) (AF3390, 1:500, Affinity), rabbit monoclonal antibody against p-p65 (Ser538) (ab76302, 1:1,000, Abcam), rabbit monoclonal antibody against IκBα (ab32518, 1:2,000, Abcam), rabbit monoclonal antibody against p- IκBα (ab133462, 1:10,000, Abcam), or rabbit monoclonal antibody against GAPDH (19F00411, 1:1,000, ZSGB-BIO). Subsequently, the membranes were incubated with the HRP-labeled secondary antibody (1:10,000, Beyotime) for exposure using an ultra-sensitive multifunctional imager (GE, United States). ImageJ software was used to analyze the gray value of the obtained image.

### Statistical analysis

All data are presented as means ± SEM. A two-tailed Student’s *t*-test was used to compare the differences between two groups. One-way ANOVA or two-way ANOVA was used to compare the differences among multiple groups. Statistical analyses were performed by SPSS 23.0 software. A value of *p* < 0.05 or *p* < 0.01 was considered to be statistically significant.

## Results

### Gut microbial lipopolysaccharide with pro-atherogenic properties is increased in atherosclerotic mice

**FIGURE 1:**
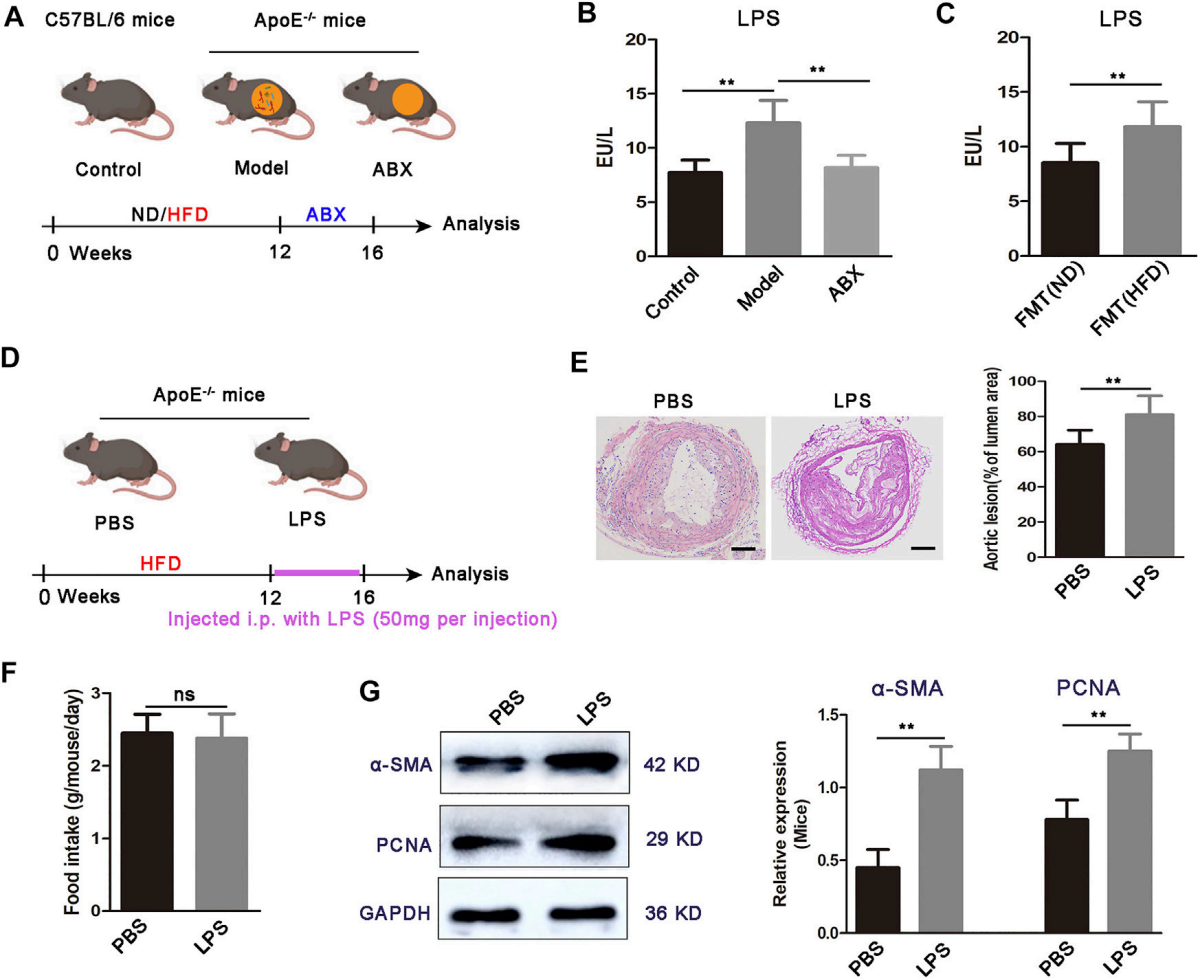
Increased LPS concentration and LPS administration exacerbates atherosclerosis development in ApoE^−/−^ mice. **(A)** Scheme of the experiment. ApoE^−/−^ mice fed with HFD for 16 weeks were maintained for the last 4 weeks of HFD feeding on HFD or broad-spectrum antibiotics (ABX) containing water. C57BL/6 mice were fed a normal diet (ND) for 16 weeks as a control group. **(B)** Concentration of LPS in the serum for C57BL/6 mice (control, *n* = 7), ApoE^−/−^ mice fed on HFD (model, *n* = 6), and ApoE^−/−^ mice fed on antibiotics containing water (ABX, *n* = 6). **(C)** Concentration of serum LPS in FMT (ND) and FMT (HFD) groups (*n* = 7). **(D)** Scheme of the experiment. ApoE^−/−^ mice were administered with PBS or LPS every 3 days for the last 4 weeks of HFD feeding. **(E)** Representative images of aortic sections and quantification of the atherosclerosis lesion size from ApoE^−/−^ mice treated with PBS or LPS. Scale bar: 100 μm. **(F)** Average daily food intake for the PBS and LPS groups of mice (*n* = 7). **(G)** Representative Western blots for α-SMA, PCNA, and GAPDH in aortas from ApoE^−/−^ mice upon treatment with PBS or LPS. Data are shown as mean ± SEM. Statistical significance was calculated using one-way ANOVA (B) or two-tailed Student’s *t-*tests (C-G). (ns: no significant difference; *p < 0.05 and **p < 0.01).

Evidence demonstrated that HFD feeding altered gut microbial composition and increased the abundance of the gram-negative bacteria and the level of gut microbial metabolite LPS (Su et al., 2022). In agreement with previous results, we observed a significantly higher fecal *Proteobacteria* level in ApoE^−/−^ mice, and the increase was 7.7-fold higher than the control group after 12 weeks of HFD (unpublished data). Gut microbial LPS, the core component of the outer membrane of gram-negative bacteria, is recognized as one of the main culprits inducing atherosclerosis-related vascular inflammation (Stephens and von der Weid, 2020). Indeed, we found elevated LPS in the serum of atherosclerosis mice (Figures 1A,B). Moreover, microbiota depletion by antibiotics reduced the level of LPS, while fecal transplant from HFD-fed atherosclerotic donors increased it in ND-fed ApoE^−/−^ mice (Figure 1C).

We then examined the effects of gut microbial LPS on plaque formation in atherosclerosis mice (Figure 1D). We used LPS to continuously stimulate mice for 4 weeks and found that atherosclerotic lesion size was increased in LPS mice compared to the PBS group (Figure 1E). Moreover, we did not observe significant differences in food intake between PBS mice or LPS mice (Figure 1F), inferring that LPS promoted plaque formation was not due to increased food intake. Abnormal proliferation of VSMCs is a critical determinant in the pathogenesis of atherosclerosis (Lin et al., 2021). Therefore, we investigated whether mice aortic VSMC shows an increased proliferative response in the LPS group. As Figure 1G showed, α-SMA and PCNA expressions are significantly increased in the aortic segments from LPS mice, compared with the PBS group. Taken together, these results showed that the high concentration of LPS potentiates specific HFD-induced atherosclerosis progression, with abnormal VSMC proliferation.

Overall, these data suggested a mechanistic connection between expansion of gram-negative bacteria, elevated bacteria-derived LPS in the serum, and VSMC proliferation in the aorta.

### Osteopontin upregulation links microbial lipopolysaccharide to vascular smooth muscle cell proliferation

**FIGURE 2:**
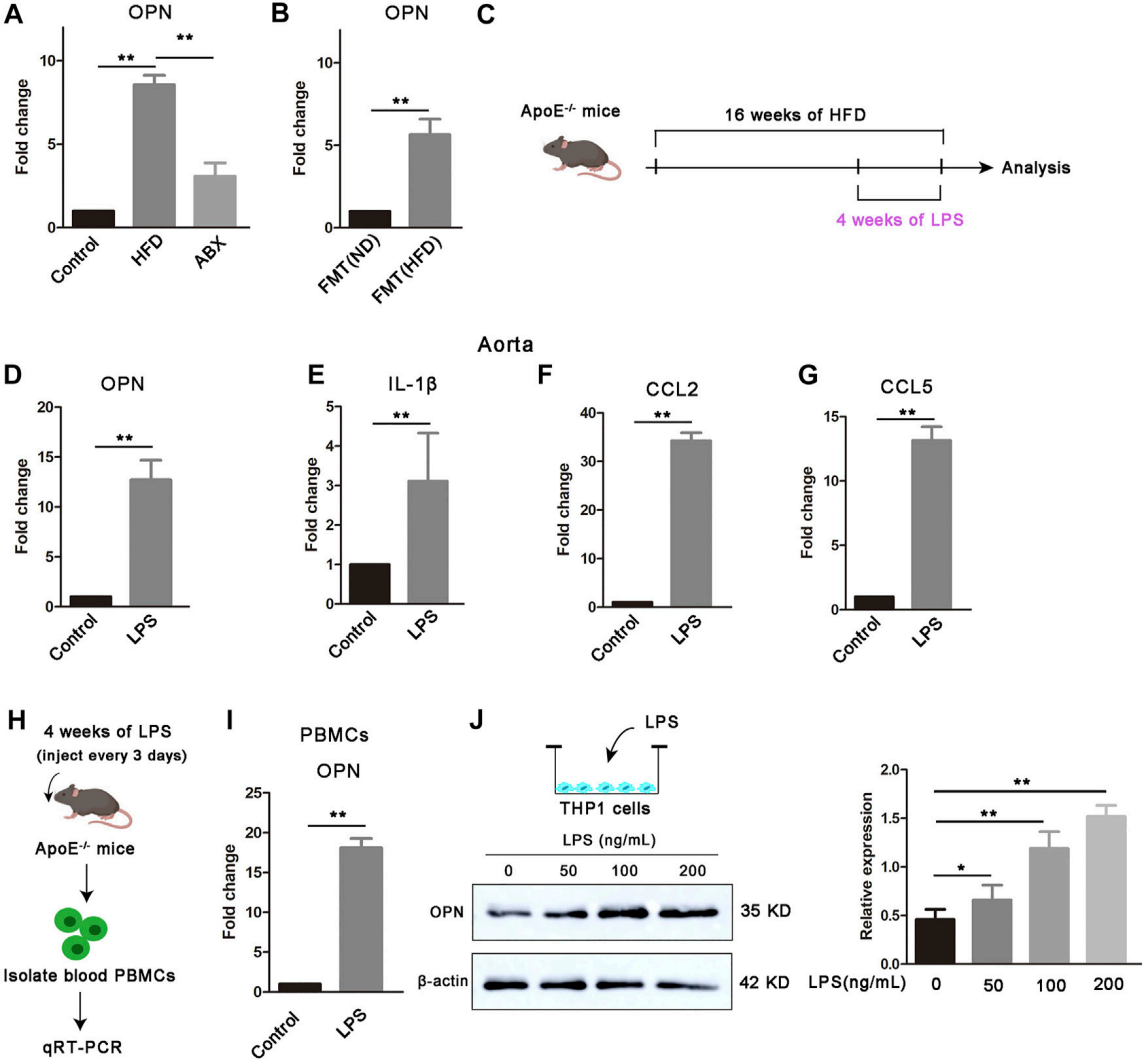
Gut microbial LPS controls the expression of OPN. **(A)** OPN gene expression in the aortas of mice (*n* = 6). **(B)** OPN gene expression in the aortas of FMT (ND) and FMT (HFD) groups (*n* = 6). **(C)** Scheme of the experiment. ApoE^−/−^ mice were administered with PBS control or LPS every 3 days for the last 4 weeks of HFD feeding. **(D–G)** OPN, IL-1β, CCL2, and CCL5 gene expressions in the aortas of ApoE^−/−^ mice treated with LPS or PBS control (*n* = 6). **(H)** Scheme of the experiment. PBMCs were extracted from ApoE^−/−^ mice after being treated with LPS and **(I)** OPN gene expression in PBMCs (*n* = 6). **(J)** Expression of OPN in THP-1 cells treated with different concentrations of LPS (0, 50, 100, and 200 ng/ml) for 24 h. Results are shown as mean of three independent experiments. Data are shown as mean ± SEM. (ns: no significant difference; *p < 0.05 and **p < 0.01).

Multi-cell information exchange occurs in the circulating blood and aortic vessels to support VSMC proliferation. To further determine how the gut microbial LPS affect VSMC proliferation in aortas, we found that OPN, the major gene encoding OPN, is highly upregulated in the aorta of atherosclerotic mice (Figure 2A). Furthermore, depletion of the microbiota by antibiotics resulted in a significant downregulation of OPN in atherosclerotic mice (Figure 2A), whereas microbiota from atherosclerotic donors increased gene expression of OPN (Figure 2B), suggesting that a specific microbiota is available in atherosclerotic mice capable of inducing OPN. OPN is a secreted inflammatory protein, which is related to the proliferation of various cells and recommended to play a role in atherosclerosis (Shevde and Samant, 2014). New evidence showed that OPN was highly expressed in aortic macrophages, while moderately expressed in macrophages from other tissues (Qin et al., 2018).

The data we have obtained have proven that LPS can significantly promote the proliferation of VSMCs and aggravate atherosclerosis. Next, we found that LPS upregulated gene expression levels of OPN, IL-1β, CCL2, and CCL5 in the aorta after 4 weeks of continuous LPS stimulation (Figures 2C–G). We further found that LPS enhanced the gene expression of OPN in circulating PBMCs (Figures 2H,I). To confirm that LPS was an important trigger of OPN in monocytes, we stimulated THP-1 cells with LPS at different concentrations and found that the protein expression level of OPN was significantly upregulated under LPS stimulation (Figure 2J). Taken together, we hypothesized that these upregulated OPN after LPS stimulation might act as a bridge between blood cells and vascular wall to mediate cross-talk and thus regulate the proliferation of VSMCs.

### Osteopontin accelerates the proliferation of vascular smooth muscle cells *via* the αvβ3/NF-κB pathway

**FIGURE 3:**
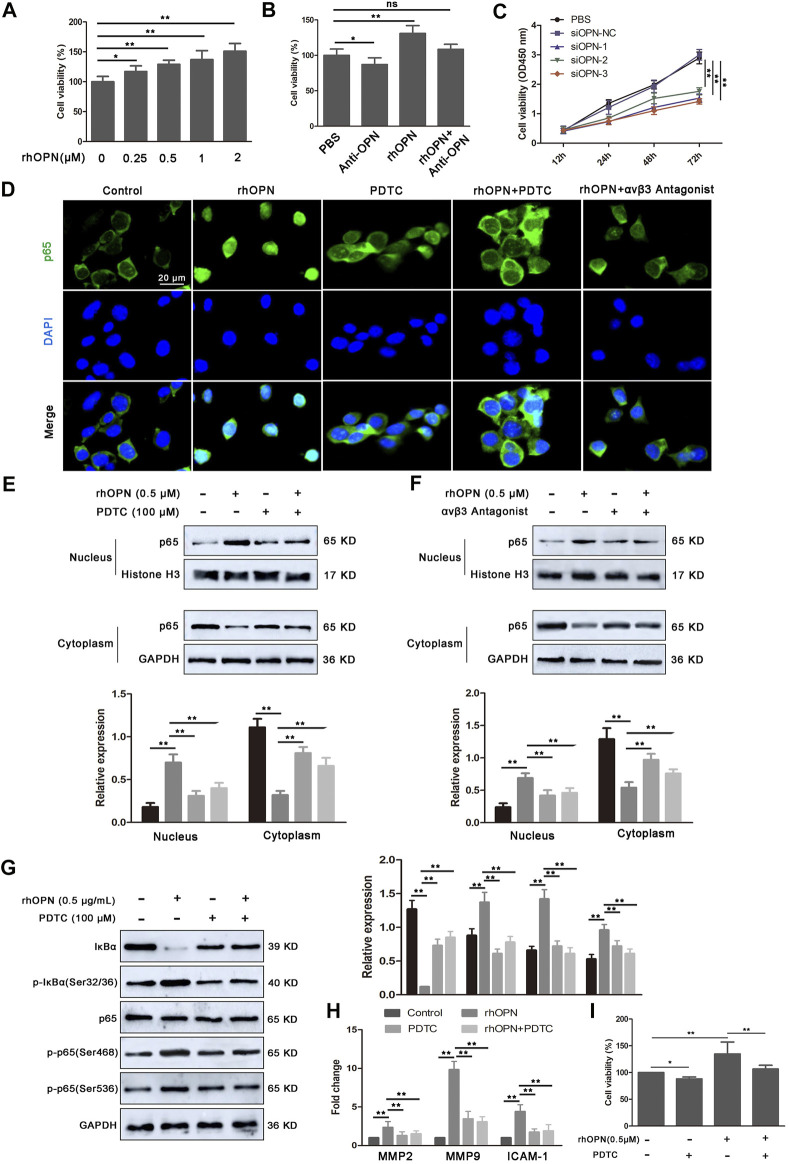
Recombinant human OPN (rhOPN) promotes VSMC proliferation *via* the αvβ3/NF-κB signal pathway. **(A,B)** rhOPN increased the cell viability of VSMCs in a dose-dependent manner, while OPN-neutralizing antibody (5 μg/ml) reversed the increase of VSMC viability induced by rhOPN. **(C)** Knockdown of OPN significantly attenuated proliferation of VSMCs. **(D–F)** Immunofluorescence assays and Western blot analysis were used to assess the location of p65 in VSMCs (scale bar: 20 μm). **(G)** Expression level of proteins related to the NF-κB signaling pathway in VSMCs, following treatment with rhOPN. **(H)** Effect of rhOPN on the gene expression of related transcription factors (MMP2, MMP9, and ICAM-1) in VSMCs. **(I)** OPN-induced VSMC proliferation was partially neutralized by pretreatment with PDTC. Data are shown as mean ± SEM. (ns: no significant difference; *p < 0.05 and **p < 0.01).

When microbiota metabolites like LPS can potentially drive atherosclerosis directly, they can also regulate inflammatory intermediates essential for disease pathogenesis. Based on the data obtained, we found strong upregulation of OPN in circulating PBMCs of atherosclerotic mice. To evaluate whether OPN was critical for the proliferation of VSMCs, recombinant human OPN (rhOPN) was added to the culture medium of VSMCs. Indeed, rhOPN promoted the proliferation of VSMCs in a dose-dependent manner, while the addition of OPN-specific neutralizing antibody inhibited the proliferation of VSMCs (Figure 3B). Meanwhile, silencing of OPN by siRNA in VSMCs partially decreased the proliferation of VSMCs (Figure 3C). These results demonstrated that OPN promotes VSMC proliferation.

Next, we further determined the molecular mechanism of OPN promoting VSMC proliferation. In tumor cells, OPN promoted cell proliferation through the NF-κB signaling pathway. Therefore, we investigated whether OPN-induced proliferation of VSMCs was also mediated by the NF-κB signaling pathway. Therefore, we evaluated the effect of rhOPN on nuclear translocation of p65. Fluorescence microscopy analysis showed complete nuclear translocation of p65 after rhOPN stimulation in VSMCs (Figure 3D). Moreover, pyrrolidine dithiocarbonate (PDTC), a selective inhibitor of NF-κB, reversed the promotive effect of rhOPN on p65 nuclear translocation in VSMCs (Figure 3D). In order to further confirm the promoting effect of OPN on p65 translocation in VSMCs, we extracted nuclear and cytoplasmic proteins, respectively. Western blot analyses showed that, in VSMCs without pre-treatment, p65 was mainly distributed in the cytoplasm, while the expression level of p65 in the nucleus was significantly upregulated after rhOPN stimulation. Pretreatment of VSMCs with PDTC or αvβ3 antagonist resulted in cytoplasmic accumulation of p65 (Figures 3E,F). In addition, rhOPN promoted the phosphorylation of IκBα and p65 in VSMCs, while PDTC inhibited this process (Figure 3G). These data indicated that OPN activates the αvβ3/NF-κB signaling pathway in VSMCs.

In order to explore the effect of p65 nuclear transfer induced by rhOPN on downstream transcription factors, we selected three transcription factors (MMP2, MMP9, and ICAM-1), which are closely related to NF-κB (Rangaswami et al., 2006; Ahmed and Kundu, 2010). Our results showed that the gene expression levels of MMP2, MMP9, and ICAM-1 were significantly upregulated after rhOPN stimulation, and PDTC reversed the upregulation of transcription factor expression in VSMCs induced by rhOPN (Figure 3H). We then investigated the effect of the OPN-induced αvβ3/NF-κB signaling pathway on the proliferation of VSMCs. Functionally, it was found that the cell viability of VSMCs after rhOPN treatment was significantly increased, while PDTC pretreatment reversed the promoting effect of rhOPN on VSMC proliferation (Figure 3I). These data suggested that OPN does promote VSMC proliferation by activating the αvβ3/NF-κB signaling pathway.

### Osteopontin is required for lipopolysaccharide-stimulated THP-1 cell–vascular smooth muscle cell crosstalk and promotes the proliferation of vascular smooth muscle cells

**FIGURE 4:**
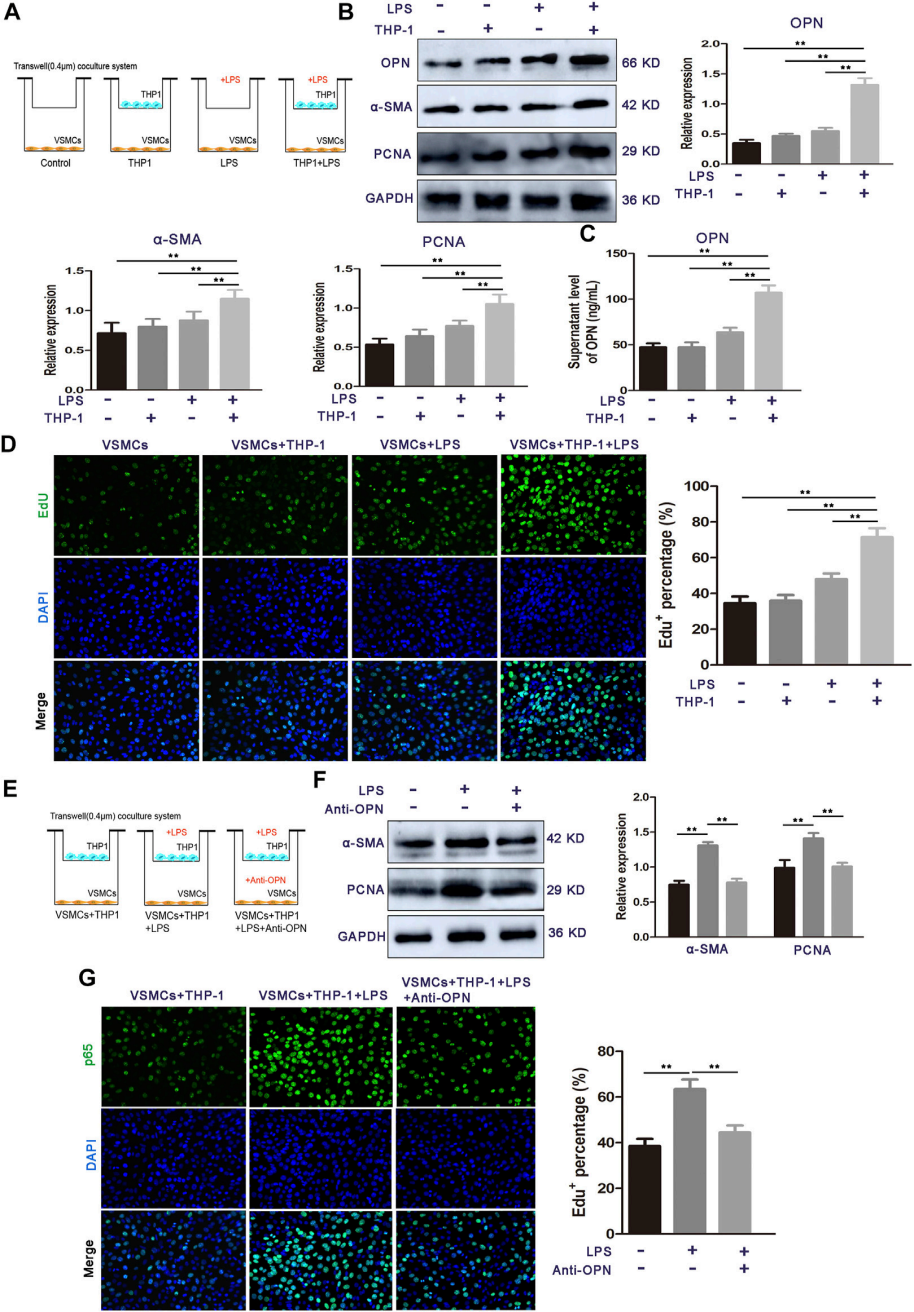
OPN is required for LPS-stimulated THP-1 cell-induced VSMC proliferation. **(A)** Schematic representation for representing the experimental procedures. **(B)** Cell proliferation capacity of VSMCs alone or co-cultured with monocytes (LPS-stimulated THP-1 cells or THP-1 cells alone) was determined by Western blot. **(C)** ELISA assay of OPN protein secretion of VSMCs. **(D)** Cell proliferation capacity of VSMCs alone or co-cultured with monocytes (LPS-stimulated THP-1 cells or THP-1 cells alone) was determined by Edu staining assay. **(E)** Schematic representation for representing the experimental procedures. **(F,G)** Western blot and EdU staining assay of VSMCs alone, THP-1 cells-co-cultured VSMCs, and OPN-depleted THP-1 cells-co-cultured VSMCs. Data are shown as mean ± SEM. (ns: no significant difference; *p < 0.05 and **p < 0.01).

Since the microbial LPS upregulated OPN in circulating blood mononuclear cells, in addition, rhOPN promotes the proliferation of VSMCs, we speculated that OPN might be involved in the signal transduction between THP-1 cells and VSMCs. To identify the THP-1 cell-derived factor in the co-culture system, which promotes the proliferation of VSMCs, we established a co-culture system of THP-1 cells and VSMCs *in vitro* using a Transwell chamber with a 0.4-μm porous membrane (Figure 4A) and found that the protein levels of OPN, α-SMA, and PCNA were prominently upregulated in co-cultured VSMCs compared to other groups (Figure 4B). ELISA assay further showed that the secretion of OPN in the co-culture system was significantly increased by LPS stimulation (Figure 4C). Similar results were obtained in EdU staining (Figure 4D). These results suggest that LPS-stimulated THP-1 cells mainly contribute to the pro-proliferative effect of VSMCs in the co-culture system.

To determine whether OPN is involved in these communication media between THP-1 cells and VSMCs, an OPN neutralizing antibody was used to confirm THP-1-induced VSMC proliferation, which was achieved using OPN (Figure 4E). After the OPN neutralizing antibody was applied in the co-culture medium, the protein expressions of α-SMA and PCNA were decreased (Figure 4F). Moreover, EdU staining showed that treatment of OPN neutralizing antibody significantly inhibited the proliferation of VSMCs co-cultured with LPS-stimulated THP-1 cells (Figure 4G). These results demonstrate that THP-1 cell-derived OPN is one of the major cytokines that may mediate the interplay between LPS stimulated THP-1 cells and VSMCs.

### Heightened level of serum osteopontin is correlated with lipopolysaccharide in patients with atherosclerosis

**FIGURE 5:**
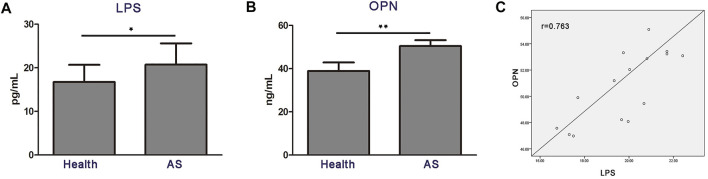
High circulating levels of OPN are associated with the increased level of LPS in patients with atherosclerosis (*n* = 15). **(A,B)** Levels of LPS and OPN in the blood of healthy controls (Health) and patients with atherosclerosis. **(C)** Scatter plots reflecting the correlation of LPS with OPN in the blood of atherosclerosis patients. Data are shown as mean ± SEM. (ns: no significant difference; *p < 0.05 and **p < 0.01).

Next, we explored whether the aforementioned findings could be recapitulated in atherosclerosis patients. Indeed, LPS and OPN concentrations in the serum of atherosclerosis patients were significantly higher than those in the age-matched healthy counterparts (Figures 5A,B). Moreover, the levels of serum LPS and OPN in patients with atherosclerosis revealed a significant positively correlation (*r* = 0.763; *p* < 0.01) (Figure 5C). These results strongly supported the idea that gut microbial LPS can influence the expression of OPN in circulating blood, thus promoting the progression of disease in patients with atherosclerosis.

### Pae exhibits ameliorative effects on atherosclerosis progression

**FIGURE 6:**
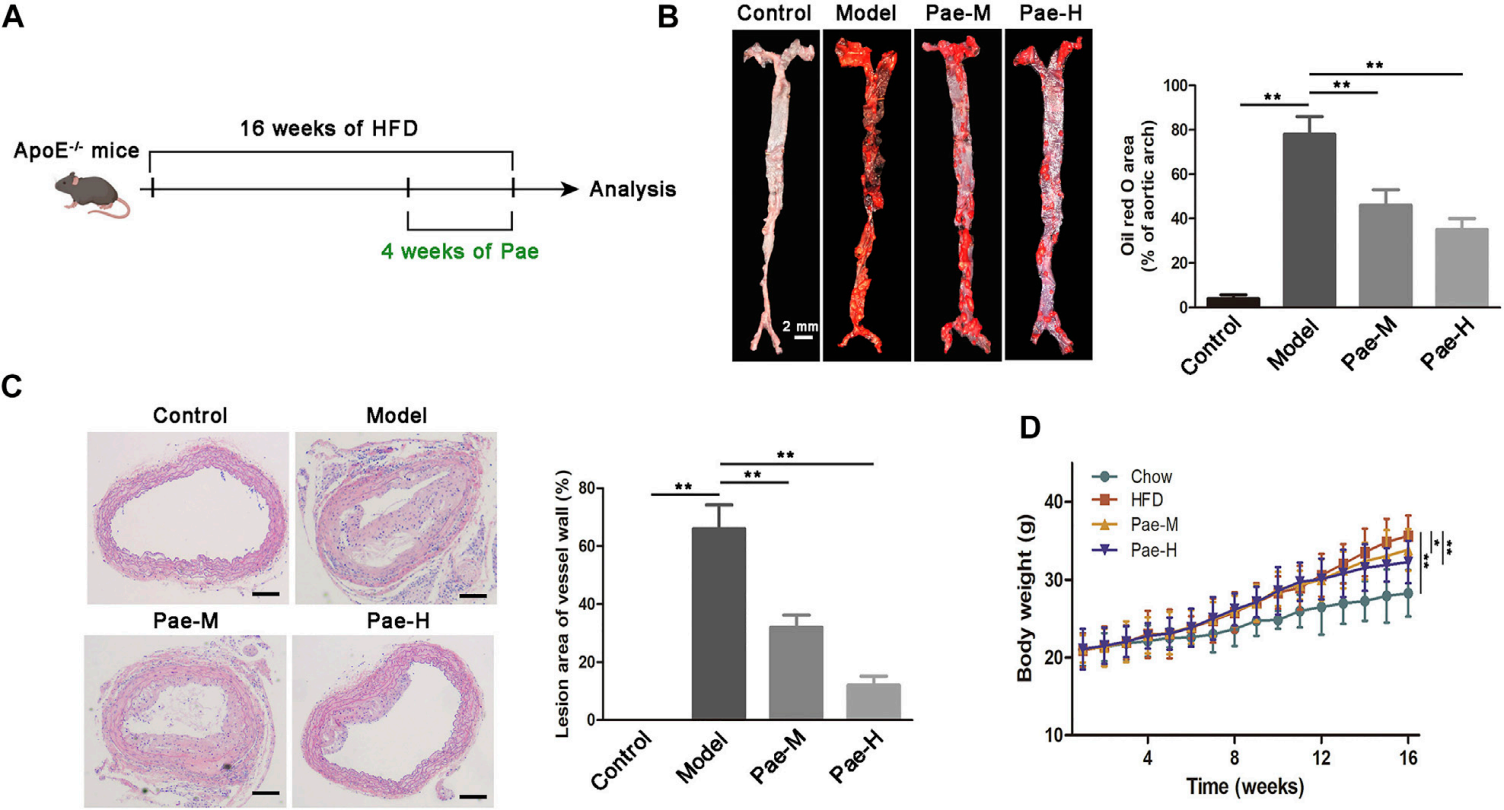
Effects of Pae on plaque formation in atherosclerotic mouse models. **(A)** Scheme of the experiment. ApoE^−/−^ mice were administered with Pae every day for the last 4 weeks of HFD feeding. **(B)** Images of ORO staining showing *en face* total aortas obtained from mice in control, model, and Pae groups. Scale bar: 2 mm. **(C)** Aortic atherosclerosis lesion of control, model, and Pae groups’ mice were stained with hematoxylin and eosin (HE). Scale bar: 100 μm. **(D)** Pae reduced the body weight of atherosclerotic mice. Data are shown as mean ± SEM. (ns: no significant difference; *p < 0.05 and **p < 0.01).

The new mechanism of the involvement of the gut-vascular axis in the atherosclerotic process triggered by gut microbial LPS revealed herein may indicate the therapeutic significance of gut microbiota intervention. To assess the effect of Pae on atherosclerosis, 5-week-old male ApoE^−/−^ mice were fed HFD for 12 weeks and then supplemented with Pae (40, and 20 mg/kg/day) for 4 weeks (Figure 6A). As expected, compared with the control group, HFD-fed ApoE^−/−^ mice showed significant increases in body weight and plaques in the aortic vessels (Figures 6B–D). Intervention of Pae notably attenuated the increase in body weight and inhibited the formation of plaques in atherosclerosis mice (Figures 6B–D). These data demonstrated that Pae may be important in regulating the progression of atherosclerosis. Recently, Pae has shown diverse modulatory effects on bacteria (Guo et al., 2020). Our previous data also confirmed that Pae played an anti-atherosclerosis effect by regulating the gut microbiota, but we are interested in exploring the underlying mechanism.

### Pae inhibits vascular smooth muscle cell proliferation in a gut microbiota-dependent manner

**FIGURE 7:**
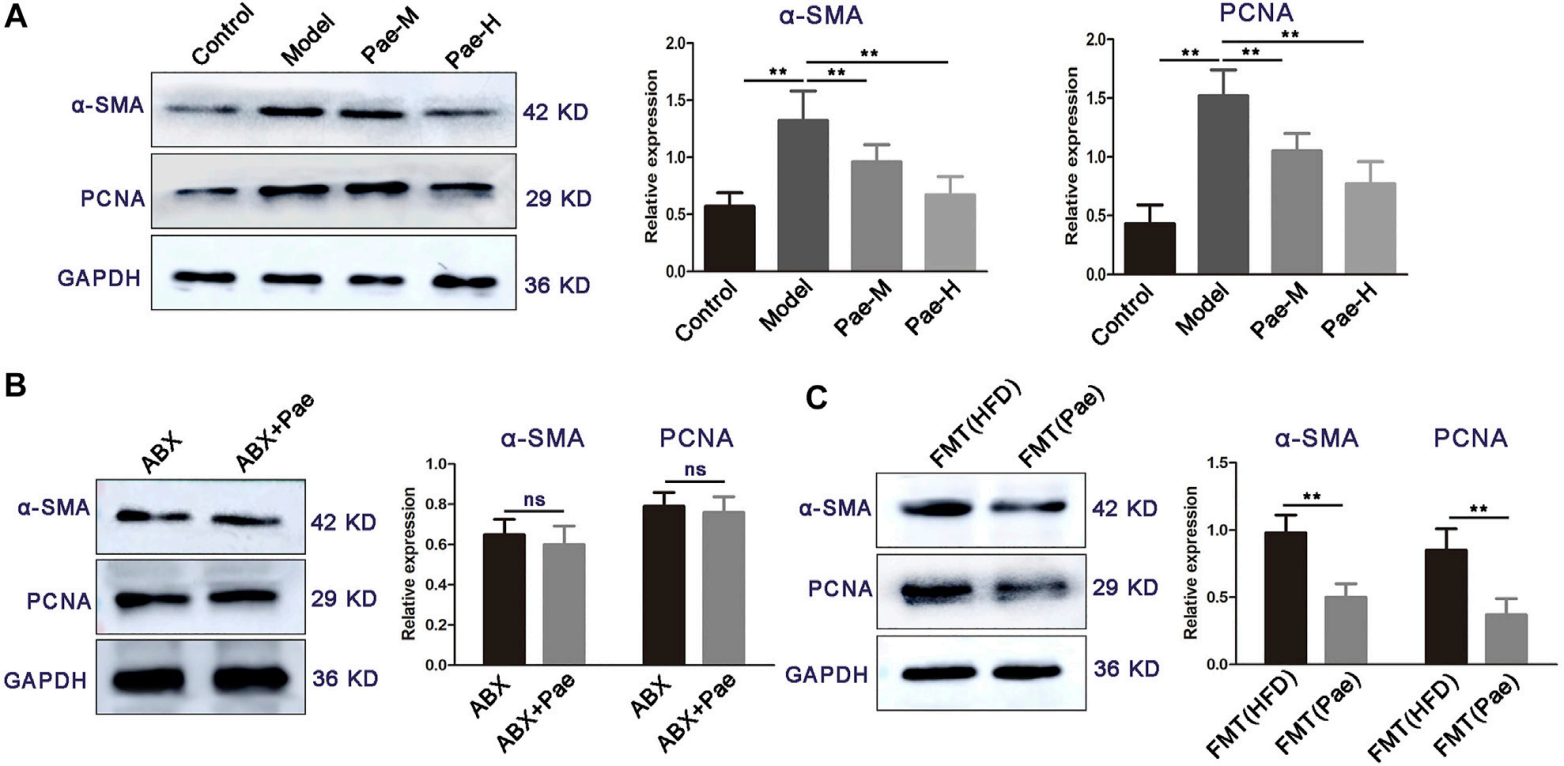
Pae inhibits VSMC proliferation in a gut microbiota-dependent manner. **(A)** Expressions of α-SMA and PCNA of the control and atherosclerotic mice treated daily with solvent (0.5% CMC-Na) or Pae for 4 weeks. **(B)** Effect of Pae against VSMC proliferation disappeared after gut-microbiota depletion. **(C)** Fecal microbiota from Pae-treated mice inhibited the proliferation of VSMCs. Data are shown as mean ± SEM. (ns: no significant difference; *p < 0.05 and **p < 0.01).

Aberrant proliferation of VSMCs promotes plaque formation in aortic vessels. We found a significant reduction in proliferation-related protein (α-SMA and PCNA) expression in the aortic of atherosclerosis mice treated with Pae compared with the HFD group (Figure 7A). Our recent studies found that Pae delayed the progression of atherosclerosis in ApoE^−/−^ mice by remodeling the homeostasis of the gut microbiota. Therefore, we next explore whether the inhibitory effect of Pae on the proliferation of VSMCs is related to the gut microbiota. Intriguingly, no significant difference in the expression of proliferation-related protein (α-SMA, and PCNA) was found between the ABX and ABX + Pae groups (Figure 7B). Furthermore, to investigate whether Pae-altered microbiota could contribute to the proliferation of VSMCs, we carried out experiments on fecal bacteria transplantation in mice. Our results further showed that horizontal fecal transfer from mice fed with Pae (FMT(Pae) group) reduced protein expression of α-SMA and PCNA compared with faecal transfer from HFD-fed mice (FMT (HFD) group) (Figure 7C). These data suggest that Pae inhibits aberrant proliferation of VSMCs in ApoE^−/−^ mice, potentially by modulating the gut microbiota.

### Pae treatment altered gut microbiota composition leading to decreased gut microbial lipopolysaccharide production

**FIGURE 8:**
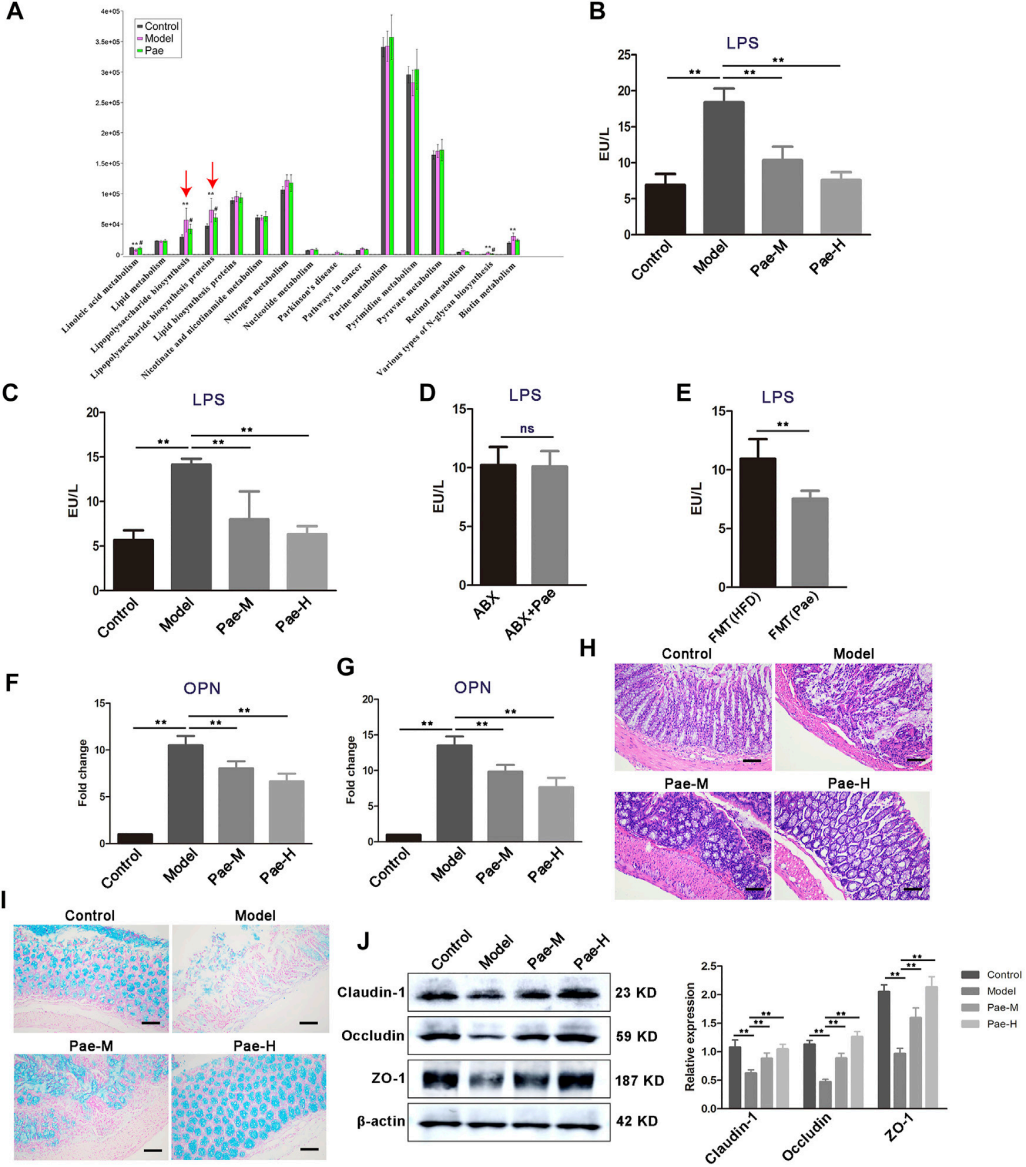
Pae alters the gut microbial LPS biosynthetic pathway and disrupted the LPS-mediated pathological pathway. **(A)** PICRUSt analysis in the KEGG pathway. Functional predictions for the fecal microbiome of each group. **(B)** Fecal LPS levels in atherosclerotic mice from each group. **(C)** Serum LPS level in atherosclerosis mice from each group. **(D)** Effects of Pae on serum LPS levels in atherosclerotic mice after gut microbiota clearance. **(E)** Pae reduced the LPS level in the serum of atherosclerotic mice by regulating gut microbiota. **(F,G)** OPN gene expression in PBMCs and aortas, respectively. **(H)** Representative HE staining of colon tissue sections from each group. Scale bar: 100 μm. **(I)** Alcian blue staining on colon tissue sections from each group. Representative images are shown. Scale bar: 100 μm. **(J)** Expression of tight junction (claudin-1, occludin, and ZO-1) proteins in the colon. Data are shown as mean ± SEM. (ns, no significant difference; *p < 0.05 and **p < 0.01).

In our previous study, 16S rDNA gene sequencing revealed changes in gut microbiota composition in atherosclerosis mice after Pae treatment, especially the significant downregulation of the abundance of gram-negative bacteria. In order to find the key pathway of Pae regulating gut microbiota to exert the antiatherosclerotic effect, we used PICRUSt software to perform functional prediction analysis. The results showed that the “lipopolysaccharide biosynthesis proteins” and “lipopolysaccharide biosynthesis pathways” were significantly changed in atherosclerotic mice after Pae intervention (Figure 8A).

LPS from pathogenic bacteria is a key link between gut microbiota and systemic low-grade inflammation and is now recognized as one of the main culprits inducing vascular inflammation. Therefore, we focus on the regulation of Pae on gut microbial LPS in the next research. We found that the concentration of LPS was significantly higher in the feces of atherosclerosis mice than those of the control group, and Pae treatment significantly reduced the concentration of LPS, after 4 weeks of Pae treatment (Figure 8B). In addition, serum level of LPS in atherosclerosis mice were also significantly reduced after Pae treatment (Figure 8C), and there was no significant difference in the level of serum LPS between ABX and ABX + Pae groups (Figure 8D). Furthermore, LPS levels in the serum of recipient mice receiving faecal bacteria from Pae treated mice were significantly reduced (Figure 8E). OPN released from THP-1 cells and peripheral blood mononuclear cells after LPS-stimulation have been shown to promote proliferation of VSMCs in our obtained data. We examined the expression level of OPN in PBMCs and found that Pae treatment inhibited the mRNA expression of OPN (Figure 8F). Furthermore, we found the same result in the aortic tissue of atherosclerosis mice (Figure 8G).

Given that HFD feeding may affect the gut barrier by increasing the gut LPS content and subsequently lead to further release of bacterial LPS into the circulation, we examined whether Pae modulates gut barrier, while disorganized glands, decreased goblet cells, and thinning of the mucus layer were further aggravated in the colon tissue of atherosclerosis mice compared with the control group (Figures 8H,I). In addition, the expression of tight junction proteins (claudin-1, occludin, and ZO-1) was significantly downregulated in atherosclerosis mice (Figure 8J), indicating that the tight junctions structure was disrupted and that the tight junctions structure was disrupted. These effects were reversed by Pae (Figures 8H,I). These findings suggest that Pae may improve gut barrier integrity in atherosclerosis mice.

## Discussion

The main findings of our study were as follows. Gut microbial LPS had a close relationship with the proliferation of VSMCs in atherosclerosis mice. Furthermore, we revealed that the activation of circulating monocytes (over-expression of OPN) is required for LPS to promote VSMC proliferation. Mechanistically, LPS aggravated VSMC proliferation by promoting OPN-mediated THP-1 monocytes-VSMC crosstalk. Finally, Pae decreased the concentration of LPS in the feces and serum, reduced the expression of OPN in PBMCs, and reduced VSMC proliferation in the aorta. Our study suggested that Pae might provide an attractive antiatherosclerotic strategy targeting gut microbiota and is worthy of future study.

Although chronic inflammation caused by circulating LPS has been established to predict accelerated atherosclerosis (Irazoqui, 2020), the underlying mechanisms of how LPS aggravates the development of atherosclerosis are largely unknown. LPS is a breakdown product, which is present on the outer membrane of gram-negative bacteria (Stephens and von der Weid, 2020). Our recent study found a disturbance of gut microbiota in atherosclerosis mice, especially the abundance of gram-negative bacteria is significantly increased, providing a link to explain why gut microbiota disorder aggravates the pathological development of atherosclerosis. Here, we observed significant changes in LPS, and increased LPS production was further elevated in recipient mice that received feces from atherosclerosis mice, implying that the microbiota-LPS pathway may be particularly important in VSMC proliferation and atherosclerosis progression.

Gut microbial LPS plays an important role in atherosclerosis-related vascular inflammation. Indeed, increased aortic inflammation in atherosclerosis mice is seen, and we found strong upregulation of OPN in circulating PBMCs. OPN in the aorta of atherosclerotic mice is known to be regulated by gut flora and its metabolites (Cho et al., 2009; Sharon et al., 2015; Lok and Lyle, 2019; Irazoqui, 2020). In present study, we found that the level of OPN was higher in the serum of atherosclerosis patients, and its level was positively correlated with LPS. Accumulating evidence has indicated that OPN can be produced by several types of immune cells, including activated T cells, macrophages, dendritic cells (DCs), and OPN is a matricellular cytokine highly expressed by bone marrow-derived myelomonocytes, regulates immune cell communication, and response to vascular damage (Rentsendorj et al., 2018; Juanola et al., 2021). OPN secreted by macrophages is an important regulator of inflammation in atherosclerosis (Cho et al., 2009). Hansakon et al. (2021) reported that OPN was predominantly expressed in alveolar macrophages during *Cryptococcus neoformans* infection. Our data reveal that LPS upregulated the expression of OPN in circulating PBMCs. Thus, the induction of monocyte/macrophages OPN by gut microbial LPS may be associated with VSMCs proliferation during atherosclerosis progression (Hansakon et al., 2021; Roy-Chowdhury et al., 2021). However, in present study, the mechanism by which LPS promoted the proliferation of VSMCs in aorta is yet to be fully uncovered, and regulation of the OPN of circulating monocytes is a possibility to explore. The answer to these questions, as well as how the OPN mediates the crosstalk between circulating monocyte and VSMCs, is critical to optimize the discovery of novel therapeutic strategies.

The complex communication between monocyte/macrophages and VSMCs within the vascular microenvironment affects the atherosclerosis development. We found that OPN was significantly upregulated in the VSMCs co-cultured with LPS stimulated THP-1 cells. Although the proliferation-promoting role of OPN in various cancer cells has been widely accepted, the role of OPN in VSMCs proliferation remains to be not fully studied. The RGD domain of OPN can bind to the integrin receptors on the cell surface and play a biological role (Dechend et al., 2003; Singh et al., 2017). Furthermore, evidence has suggested a causative role for NF-κB in proliferation of VSMCs. In our study, PDTC and αvβ3 antagonists significantly inhibited OPN-promoted nuclear transfer of p65 (Chen et al., 2019; Kong et al., 2019). OPN contributed to VSMC proliferation *via* the integrin αvβ3/NF-κB axis. However, the question remains as to whether OPN is related to THP-1 cells-VSMC crosstalk. To answer this question, we found that LPS stimulation of THP-1 cells significantly promoted the proliferation of VSMCs, and the addition of OPN neutralizing antibodies reversed this result. However, this mechanism only partially illustrates the promoting role of OPN in the proliferation of VSMCs, and more work is needed to further clarify the role of OPN in the future.

Pae as an ancient anti-inflammatory medication has been reported to be an effective remedy for atherosclerosis (Liu et al., 2018; Shi et al., 2020). However, the specific *in vivo* targets of Pae have not been elucidated, and the poor oral bioavailability of Pae suggests that it has a longer residence time in the gut and may have a modulatory effect on gut microbiota (Chen et al., 2018). Indeed, our previous data have shown significant gut microbiota alterations induced by Pae. In present study, feces from Pae-treated atherosclerosis mice could largely mimic the inhibitory effects of Pae on VSMC proliferation. These findings provided important evidence that the effect of Pae on inhibiting aortic VSMC proliferation is primarily mediated by the gut microbiota. Based on this notion, we found that LPS biosynthesis was most pronounced in downregulated pathways in Pae-treated mice. Furthermore, we found that Pae effectively decreased the concentration of LPS in the feces and serum, and reduced VSMC proliferation in aorta, implying that the gut microbiota-LPS pathway may be particularly important for Pae to exert antiatherosclerosis effects. Pae therefore decreased the level of gut microbial LPS and distantly inhibited VSMC proliferation *via* regulation of gut barrier and inflammation, although we do not exclude an aorta-specific role of these cytokines.

## Conclusion

Overall, our data show that microbiota dysbiosis affects microbial LPS producing, leading to increased expression of OPN in circulating monocyte/macrophages and promoted VSMC proliferation. This might be the pathway, at least in part, that links microbiota dysbiosis to atherosclerosis. Because we were able to interrupt this pathological gut microbiota-related pathway by administering Pae, our study supports the concept that Pae interventions might be an effective strategy in the therapeutic arsenal to fight atherosclerosis.

## Data Availability

The original contributions presented in the study are included in the article/Supplementary Material; further inquiries can be directed to the corresponding author.

## Abbreviations

α-SMA, alpha smooth muscle actin; ABX, broad-spectrum antibiotic; ApoE, apolipoprotein E; AS, atherosclerosis; CCK-8, cell counting kit-8; CCL2, (C–C motif) ligand 2; CCL5, (C–C motif) ligand 5; DAPI, 4′,6-diamidino-2-phenylindole; FMT, fecal microbiota transplantation; HE, hematoxylin and eosin; HFD, high-fat diet; ICAM-1, intercellular adhesion molecule 1; IL-1β, interleukin 1 beta; LPS, lipopolysaccharide; MMP2, matrix metalloproteinases 2; MMP9, matrix metalloproteinases 9; ND, normal diet; NF-κB, nuclear factor-kappa B, OPN: osteopontin; Pae, paeonol; PBMCs, peripheral blood mononuclear cells; PICRUSt, phylogenetic investigation of communities by reconstruction of unobserved states; PCNA, Preventive Cardiovascular Nurses Association; PDTC, pyrrolidine dithiocarbonate; qRT-PCR, quantitative real-time PCR; rhOPN, recombinant human OPN; THP-1 cells, Tohoku Hospital Pediatrics-1 cells, VSMCs: vascular smooth muscle cells.
